# Effect of two multi-step polishing systems on surface characteristics of nanohybrid composite resins: Influence of reuse

**DOI:** 10.4317/jced.62873

**Published:** 2025-08-01

**Authors:** Ana María Restrepo, Carlos Andrés Giraldo, Ana María Torres-López, Federico Latorre-Correa, Carlos M Ardila

**Affiliations:** 1 Prosthodontics Postgraduate Program, Faculty of Dentistry, University of Antioquia, Medellín, Colombia; 2 PhD. Biomaterials Research Group, Associate Professor, Bioengineering Program, Faculty of Engineering, University of Antioquia, Medellín, Colombia; 3 Titular Professor, Prosthodontics Postgraduate Program, Faculty of Dentistry, University of Antioquia, Medellín, Colombia; 4 PhD. Titular Professor, Department of Basic Sciences, Biomedical Stomatology Research Group, Faculty of Dentistry, University of Antioquia, Medellín, Colombia; 5 Department of Periodontics, Saveetha Dental College, Saveetha Institute of Medical and Technology Sciences, SIMATS, Saveetha. University, Chennai, Tamil Nadu, India

## Abstract

**Background:**

Proper finishing and polishing are crucial for reducing surface roughness and eliminating the incompletely polymerized oxygen-inhibited layer. This study compared surface characteristics - specifically water contact angle, surface roughness, and morphology of three nanohybrid composite resins polished with two different multi-step polishing systems.

**Material and Methods:**

We evaluated Filtek Z250 (3M), Tetric N-Ceram (Ivoclar), and Spectra Smart (Dentsply), all in shade A2. Thirty-nine discs per resin (9 mm diameter × 2 mm thickness) were fabricated, stored in distilled water at 37°C for 24 hours, and pre-polished with silicon carbide paper (600, 1000, and 1200 grit). Final polishing employed two systems: the 3-step Astropol system (Ivoclar) and the 2-step ShapeGuard system (Diatech), with each system tested through six reuse cycles (three samples per resin per cycle). Surface analysis included water contact angle measurement, profilometry, scanning electron microscopy (SEM), and energy-dispersive X-ray spectroscopy (EDS).

**Results:**

The Astropol system demonstrated significantly greater hydrophobicity (107.6° ± 10.9°) than ShapeGuard (99.1° ± 9.0°). Among the tested resins, Filtek Z250 polished with Astropol showed the lowest surface roughness (0.27 ± 0.11 µm), while Spectra Smart polished with ShapeGuard showed the highest (0.78 ± 0.20 µm). Polishing system reuse showed no significant effects on results. SEM revealed no noTable morphological changes, and EDS confirmed consistently high SiO2 content across all resins.

**Conclusions:**

Both polishing system and resin composition significantly influence surface characteristics. Filtek Z250 performed optimally with both polishing systems. Further research should investigate the long-term effects of polishing system reuse.

** Key words:**Nanohybrid resins, polishing systems, surface roughness, contact angle, SEM.

## Introduction

Composite restorations have evolved significantly to meet both aesthetic and functional demands. Advances in their physical, chemical, and mechanical properties have enhanced their ability to mimic natural dentition while improving clinical longevity [1,2]. However, increased surface roughness remains a concern as it promotes bacterial plaque accumulation, increases susceptibility to secondary caries, reduces color stability, accelerates wear, and compromises structural integrity [3-5]. Studies indicate that maintaining surface roughness below 0.2 µm minimizes bacterial adhesion and extends restoration longevity [1,2,6].

Composite resins are widely used in dental restorations, with nanohybrid composites being particularly popular. Their heterogeneous filler composition combines larger particles (which improve mechanical strength) with smaller particles (which enhance filler loading, reduce polymerization shrinkage, and improve optical properties and polishability) [7-10].

Proper finishing and polishing are crucial for reducing surface roughness and eliminating the incompletely polymerized oxygen-inhibited layer. Polishing effectiveness depends on factors including the resin’s degree of conversion and filler particle characteristics. Both multi-step and simplified polishing systems have been developed to optimize smoothness, anatomical precision, aesthetics, and biocompatibility. Recently, one- and two-step systems have gained popularity due to their efficiency and ease of use. These systems typically employ silicone tips or spiral discs embedded with abrasive particles, allowing finishing, polishing, and shining in minimal steps [11-13].

Previous studies have reported varying surface roughness outcomes depending on the resin and polishing system used [3,4,13,14]. For instance, Jung *et al*. demonstrated that the Astropol system produced lower surface roughness (0.174 µm) on Premise resin compared to PoGo (0.258 µm) and OptiShine (0.274 µm) [4]. Similarly, Pierre *et al*. found superior results with the three-step Astropol system versus two-step alternatives [2], while Kormarz *et al*. confirmed these findings, favoring multi-step systems [13].

Surface roughness also varies significantly among composite resins. Filtek Supreme XT showed lower roughness (0.121 µm) than other materials including Duralfill (0.197 µm), Grandio SO (0.149 µm), and Venus Pearl (0.156 µm) [2,14,15].

Despite the widespread use of polishing systems, evidence regarding their repeated reuse remains limited. Türkün’s evaluation of the PoGo system found no significant differences in surface roughness after four uses, but observed significant increases by the fifth and sixth cycles [16]. This gap in knowledge highlights the need to investigate how wear affects polishing performance on nanohybrid composites.

Therefore, this study evaluated the surface roughness, water contact angle, and morphology of three commercially available nanohybrid resins polished with two systems: ShapeGuard (Diatech) and Astropol (Ivoclar). Additionally, it examined the effects of up to six polishing cycles on surface characteristics.

## Material and Methods

- Preparation of Resin Specimens

A total of 117 resin discs (9 mm in diameter and 2 mm in thickness) were fabricated and divided into three groups (A, B, and C), with 39 specimens per group. Each group corresponded to one of the following nanohybrid composite resins: Filtek Z250 (3M), Tetric N-Ceram (Ivoclar), and Spectra Smart (Dentsply), all in shade A2.

Specimens were prepared using a stainless-steel mold coated with glycerin to prevent resin adhesion. The composite was inserted in a single 2 mm increment using an OptraSculpt spatula (Ivoclar Vivadent). A Mylar strip was placed on the top surface, followed by compression with a glass slide to ensure uniformity.

Polymerization was performed with a Bluephase N curing light (Ivoclar Vivadent), previously calibrated using a radiometer. The device operated in high-intensity mode at 1200 mW/cm², with a wavelength range of 385–515 nm, applied for 40 seconds. The light tip was positioned perpendicular to the specimen surface at a distance of 1 mm, maintained using a custom-made positioning device. After curing, all samples were removed from the mold, labeled, and stored in distilled water at 37°C for 24 hours to simulate the moist conditions of the oral environment.

- Polishing Protocol

All specimens were pre-polished using wet silicon carbide papers (grit sizes 600, 1000, and 1200), in accordance with ISO 8486-1:1996. Each abrasive paper was used only once. Final polishing was performed under 10× magnification using a ZEISS microscope to replicate clinical polishing conditions.

Two polishing systems were used:

ShapeGuard (Diatech-Coltene): A two-step system consisting of multilayer-coated spiral tips. Step 1 (pink spiral) has medium granularity (32–69 µm), and Step 2 (blue spiral) has extra-fine granularity (4–8 µm).

Astropol (Ivoclar): A three-step system with discs: F (gray, for finishing), P (green, for polishing), and HP (pink, for high-gloss polishing). These are composed of silicon carbide, silicone rubber, and, in the HP disc, diamond particles with aluminum oxide, titanium oxide, and silicone rubber.

Polishing was performed at 10,000 rpm for 20 seconds using horizontal, unidirectional strokes without irrigation, employing an electric handpiece (W&H). Each system was used up to six times (reuses) on different samples. After polishing, specimens were rinsed under pressurized water for 10 seconds and cleaned in an ultrasonic bath (BIOWASH BIOART, São Carlos, SP, Brazil) for 2 minutes.

- Surface Characterization

Water Contact Angle:

Wettability was measured using a goniometer (OCA 15EC, Dataphysics, Filderstadt, Germany). Distilled water droplets were placed on the sample surfaces, and contact angles were measured on both sides using OCA software. Measurements were performed three times for each sample, corresponding to the 1st, 3rd, and 6th reuse cycles.

Surface Roughness:

Surface roughness (Ra) was measured with a Surtonic S128 profilometer (AMETEK Ultraprecision Technologies, Berwyn, PA). Each sample was tested three times, and Ra values (in µm) were recorded for the 1st, 3rd, and 6th cycles of polishing.

Surface Morphology and Elemental Composition:

One sample per resin and polishing cycle was prepared for scanning electron microscopy (SEM) and energy-dispersive X-ray spectroscopy (EDS). Samples were cleaned, sputter-coated with gold (Denton Vacuum Desk IV), and examined using a JEOL JSM-6490LV microscope (Fargo, North Dakota, USA) at magnifications of 500× and 5000×. Elemental surface composition was assessed with EDS.

- Statistical Analysis

Data were analyzed using IBM SPSS software. The Shapiro–Wilk test was used to verify normal distribution. Independent samples t-tests were applied to evaluate the effect of polishing system and reuse on surface roughness and wettability. One-way ANOVA was used to compare surface characteristics across different resins and reuse cycles. When ANOVA showed significant results, post hoc multiple comparisons were conducted to identify specific differences. The significance level was set at *p* < 0.05.

## Results

- Water Contact Angle

Contact angles above 90° were observed in both the polished and control samples. Statistically significant differences (*p* = 0.000) were found between the two polishing systems ( Table 1). The Astropol system (Ivoclar) exhibited higher hydrophobicity, with an average contact angle of 107.6° ± 10.9°, compared to the ShapeGuard system (Diatech), which showed 99.1° ± 9.0°. The control group displayed the lowest hydrophobicity (95.7° ± 4.4°).

Among the individual resins, Spectra Smart (Dentsply) showed the highest contact angle when polished with Astropol (110.2° ± 2.7°), while Z250XT (3M) polished with ShapeGuard had the lowest (91.5° ± 6.3°). ANOVA revealed significant differences between resins for each polishing system (*p* < 0.001).

- Surface Roughness

 Table 2 shows the mean Ra values (µm) for the three resins after polishing with Astropol and ShapeGuard, as well as the control group.

Z250XT (3M): The lowest roughness was achieved with Astropol (0.27 ± 0.11 µm), while ShapeGuard resulted in a slightly higher value (0.34 ± 0.13 µm); the control exhibited the highest roughness (0.41 ± 0.50 µm). No statistically significant differences were found between polishing systems (*p* = 0.189).

Spectra Smart (Dentsply): Polishing with ShapeGuard produced the highest roughness (0.78 ± 0.20 µm), followed by Astropol (0.63 ± 0.10 µm). The control group had a lower Ra value (0.31 ± 0.25 µm), but differences between systems were not statistically significant (*p* = 0.060).

Tetric N-Ceram (Ivoclar): A significant difference was found between systems (*p* = 0.008), with Astropol yielding lower roughness (0.37 ± 0.17 µm) compared to ShapeGuard (0.74 ± 0.32 µm). The control group exhibited intermediate roughness (0.54 ± 0.07 µm).

Overall, Astropol produced smoother surfaces across all resins (mean Ra = 0.49 ± 0.23 µm) compared to ShapeGuard (0.63 ± 0.26 µm), with the difference being statistically significant (*p* = 0.027).

Effect of Polishing System Reuse

For the Z250XT resin, no significant differences in surface roughness (Ra) were observed across the 1st, 3rd, and 6th uses within each polishing system (Astropol: *p* = 0.629; ShapeGuard: *p* = 0.962). The mean roughness values for Astropol ranged from 0.22 ± 0.08 µm (3rd use) to 0.32 ± 0.19 µm (1st use), while ShapeGuard values ranged from 0.33 ± 0.17 µm (3rd use) to 0.36 ± 0.19 µm (1st use). However, a significant difference emerged between systems at the 6th reuse (*p* = 0.029), with Astropol yielding lower roughness (0.26 ± 0.03 µm) compared to ShapeGuard (0.35 ± 0.03 µm). The overall mean roughness across all cycles was 0.27 ± 0.11 µm (Astropol) and 0.34 ± 0.13 µm (ShapeGuard) (*p* = 0.189). Unpolished controls showed higher variability (0.41 ± 0.50 µm), though direct statistical comparison was not reported.

Spectra Smart Resin ( Table 3):

A statistically significant difference was noted at the 1st polishing cycle (*p* = 0.016), with Astropol showing lower roughness (0.58 ± 0.09 µm) than ShapeGuard (0.81 ± 0.05 µm). No significant differences were found across subsequent reuses within either system (Astropol: *p* = 0.616; ShapeGuard: *p* = 0.524).

- Tetric N-Ceram Resin ( Table 4):

Although roughness values increased progressively with reuse, differences across the 1st, 3rd, and 6th cycles were not statistically significant for either system (Astropol: *p* = 0.355; ShapeGuard: *p* = 0.235). However, a significant overall difference between the systems was observed (*p* = 0.008), with Astropol yielding smoother surfaces.

- Surface Morphology (SEM Analysis)

Filtek Z250XT and Spectra Smart showed larger and more widely distributed filler particles. No major morphological differences were observed between the polishing systems for the same resin, indicating minimal surface damage from reuse.

- EDS Analysis

Energy-dispersive X-ray spectroscopy revealed that all resins had a high SiO2 content. Unique to each formulation:

Filtek Z250XT contained 19.61% zirconium.

Tetric N-Ceram presented 15% ytterbium trifluoride and 6.44% bromine.

Spectra Smart showed consistent elemental composition across cycles, without distinctive additional elements.

## Discussion

The surface roughness of composite restorations is influenced by both the intrinsic properties of the material and the finishing and polishing techniques employed [18]. Our study provides comprehensive evidence about the impact of two multi-step polishing systems on three commercial nanohybrid resins, including an evaluation of system reuse across six applications. These findings gain particular relevance when considering recent studies demonstrating the critical relationship between surface roughness and long-term clinical performance. The work by Güntekin and Tunçdemir (2024) comparing additive and subtractive manufacturing methods revealed that material composition and fabrication techniques significantly influence surface stability under functional loads [19]. Similarly, Alharbi *et al*. (2024) confirmed that multi-step polishing systems consistently achieve superior surface characteristics compared to simplified alternatives [20], reinforcing the clinical importance of proper polishing protocols.

Our control group results using Mylar strips revealed interesting variations compared to previous literature. While Patel *et al*. [15] established that Mylar-finished surfaces typically show the lowest roughness values, we observed that polished specimens - particularly those treated with the Astropol system - often achieved comparable or even superior smoothness. This discrepancy suggests that modern polishing systems can potentially surpass the performance of conventional Mylar strips, especially when considering specific resin compositions. These findings emphasize the need for clinicians to carefully select polishing protocols based on both the restorative material and the available polishing systems.

The resin composition emerged as a critical factor determining polishing outcomes in our study. The Filtek Z250 resin achieved remarkably low surface roughness (0.22 ± 0.08 µm) when polished with the Astropol system, approaching the clinically recommended threshold of 0.2 µm for minimizing bacterial adhesion [1,6,21,22]. This excellent performance likely stems from its spherical zirconia-based filler particles, which appear particularly responsive to multi-step polishing. In contrast, Tetric N-Ceram’s distinctive composition featuring smaller, densely packed fillers and bromine content produced different polishing characteristics, while Spectra Smart’s irregular filler morphology consistently resulted in higher roughness values regardless of the polishing system used.

The superior performance of the three-step Astropol system compared to the two-step ShapeGuard system aligns with the systematic review by Jaramillo-Cartagena *et al*. [23], which established that multi-step protocols generally produce smoother surfaces than simplified systems. This advantage is particularly evident in Astropol’s final high-shine step, which incorporates diamond particles with aluminum and titanium oxides for optimal surface refinement. However, as noted by Dennis *et al*. [22], certain resin-system combinations may show exceptions to this general rule, emphasizing the importance of material-specific polishing protocols in clinical practice.

Our results corroborate the findings of Hanan *et al*. [24] regarding the effectiveness of aluminum oxide-based multi-step systems, while also confirming the observations of Altınışık and Özyurt [25] about the complex interplay between resin type and polishing outcomes [26,27]. This consistency across multiple studies strengthens the evidence base for clinical decision-making regarding polishing protocols, particularly when considering the long-term performance of composite restorations.

The reuse evaluation revealed that both polishing systems maintained acceptable performance through six applications, with the Astropol system showing particular stability in maintaining surface quality with the Z250 resin. SEM and EDS analyses confirmed the absence of morphological deterioration and consistent elemental composition across reuse cycles, supporting the clinical viability of controlled system reuse. The presence of distinctive elements like zirconium and ytterbium trifluoride in certain resins may contribute to their unique polishing characteristics and performance under repeated polishing.

Among the notable strengths of this study is its comprehensive evaluation methodology, combining quantitative surface analysis with detailed morphological characterization. The rigorous experimental design, including standardized polishing protocols and multiple analytical techniques, ensures reliable and reproducible results. Furthermore, the study’s focus on system reuse addresses an important practical consideration in clinical dentistry, providing valuable data to inform cost-effective practice management.

Several limitations should be acknowledged when interpreting these findings. The *in vitro* nature of the study, while necessary for controlled evaluation, cannot fully replicate the complex oral environment where factors like thermal cycling, biofilm accumulation, and occlusal forces continuously affect restoration surfaces. Additionally, while we evaluated three representative nanohybrid composites, the findings may not directly apply to other material categories with different mechanical properties. The six-cycle reuse evaluation, though providing valuable preliminary data, may not reflect the extreme wear patterns encountered in high-volume clinical settings.

Future research directions should include investigations under more clinically relevant conditions, incorporating dynamic loading and aging protocols. Expanded material evaluations, including newer composite formulations and alternative polishing systems, would enhance the generalizability of findings. Longer-term reuse studies could provide additional insights into the economic and practical aspects of polishing system longevity in dental practice.

## Conclusions

Filtek Z250XT demonstrated superior polishability with both polishing systems, showing the importance of resin composition on surface characteristics. The three-step Astropol system consistently produced smoother surfaces than ShapeGuard, particularly for zirconia-filled resins, due to its diamond-containing final polishing stage. Both systems-maintained performance through six reuse cycles, supporting their cost-effective clinical use. However, ShapeGuard showed earlier performance decline with harder resins, suggesting the need for periodic monitoring during extended use.

These findings highlight that optimal results require matching polishing systems to specific resin types. While Astropol offers better performance and durability, ShapeGuard remains viable for shorter-term applications. Further clinical studies should evaluate long-term reuse effects.

## Figures and Tables

**Table 1:** Water contact angle values of the 3 resins subjected to 1, 3 and 6 polishing cycles.

Resine	Astropol HP-Ivoclar	ShapeGuard Diatech- Coltene		Control
	± SD	± SD	p*	± SD
Z250XT 3M	108.3±2.3	91.5±6.3	0.0001	94.0±1.6
Spectra Smart- Dentsply	110.2±2.7	103.8±6.5	0.0001	90.7±2.4
Tetric N-Ceram-Ivoclar	107.4±2.2	100.4±4.4	0.0001	100.3±2.7
p**	0.245	0.0001	---	0.0001
Total	107.6±10.9	99.1±9.0	0.000	95.7±4.4

* Compare the surface wettability of the two polishing systems for each resin (t-Student test). ** Compare the water contact angle of the three resins for each polishing system (ANOVA test).

**Table 2:** Roughness values Ra (µm) of the 3 resins according to the polishing system subjected to 1, 3 and 6 polishing cycles.

Resine	Ra (µm)			Ra (µm)
	Astropol (n=9)	ShapeGuard (n=9)		Control
	± SD	± SD	p*	± SD
Z250XT 3M	0.27±0.11A	0.34±0.13A	0.189	0.41±0.50
Spectra Smart- Dentsply	0.63±0.10B	0.78±0.20B	0.060	0.31±0.25
Tetric N-Ceram-Ivoclar	0.37±0.17A	0.74±0.32B	0.008	0.54±0.07
p**	0.0001	0.0001	---	0.438
Total	0.49±0.23	0.63±0.26	0.027	0.45±0.16

* Compare the roughness of the two polishing systems for each resin (Student’s t-test). *p*-value <0.05 = significant difference. ** Compare the roughness of the four resins for each polishing system (ANOVA test). *p*-value <0.05 = significant difference. Equal letters in superscript indicate no significant difference, while different letters indicate a significant difference.

**Table 3:** Roughness values Ra (µm) of Spectra Smart-Dentsply resin according to the number of polishes for each polishing system.

Polishing cycle	Ra (µm)			Ra (µm)
	Astropol	ShapeGuard		Control
			p	
1	0.58±0.09	0.81±0.05	0.016	---
3	0.67±0.11	0.66±0.23	0.966	---
6	0.63±0.13	0.86±0.27	0.252	---
p	0.616	0.524	---	---
Total	0.63±0.10	0.78±0.20	0.060	0.66±0.21
No polishing	---	---	---	0.31±0.25

**Table 4:** Roughness values Ra (µm) of Tetric N-Ceram- Ivoclar resin according to the number of polishes for each polishing system.

Polishing cycle	Ra (µm)			Ra (µm)
	Astropol	ShapeGuard		Control
	±	±	p	±
1	0.49±0.22	0.99±0.41	0.135	---
3	0.28±0.04	0.54±0.24	0.146	---
6	0.33±0.18	0.69±0.17	0.070	---
p	0.355	0.235	---	---
Total	0.37±0.17	0.74±0.32	0.008	0.55±0.30
No polishing	---	---	---	0.54±0.07

## Data Availability

The datasets used and/or analyzed during the current study are available from the corresponding author.

## References

[1] Komalsingsakul A, Klaophimai A, Srisatjaluk RL, Senawongse P (2019). Effect of the surface roughness of composite resins on the water contact angle and biofilm formation. Dent J (Basel).

[2] Pierre LS, Martel C, Crépeau H, Vargas M (2019). Influence of polishing systems on surface roughness of composite resins: Polishability of composite resins. Oper Dent.

[3] Daud A, Gray G, Lynch CD, Wilson NHF, Blum IR (2018). A randomized controlled study on the use of finishing and polishing systems on different resin composites using 3D contact optical profilometry and scanning electron microscopy. J Dent.

[4] Jung M, Klimek J, Eichelberger K (2007). Surface geometry of four nanofiller and one hybrid composite after one-step and multiple-step polishing. Oper Dent.

[5] Lazarchik DA, Hammond BD, Sikes CL, Looney SW, Rueggeberg FA (2007). Hardness comparison of bulk-filled/transtooth and incremental-filled/occlusally irradiated composite resins. J Prosthet Dent.

[6] Bilgili D, Dündar A, Barutçugil Ç, Tayfun D, Özyurt ÖK (2020). Surface properties and bacterial adhesion of bulk-fill composite resins. J Dent.

[7] Ferracane JL (2010). Resin composite-State of the art. Dent Mater.

[8] Da Costa J, Ferracane JL, Paravina RD, Mazur RF, Roeder L (2007). The effect of different polishing systems on surface roughness and gloss of various resin composites. J Esthet Restor Dent.

[9] Anusavice KJ, Shen C, Rawls HR (2013). Mechanical properties of dental materials. In: Phillips’ Science of Dental Materials. 12th ed.

[10] 3M. Filtek™ Supreme XTE Universal Restorative System Technical Profile.

[11] Gonçalves MA, Teixeira VCF, Rodrigues SSMFG, de Oliveira RSMF, Salvio LA (2012). Evaluation of the roughness of composite resins submitted to different surface treatments. Acta Odontol Latinoam.

[12] Madhyastha PS, Naik DG, Srikant N, Kotian R, Bhat K (2015). Effect of finishing/polishing techniques and time on surface roughness of silorane- and methacrylate-based restorative materials. Oral Health Dent Manag.

[13] Korkmaz Y, Ozel E, Attar N, Aksoy G (2008). The influence of one-step polishing systems on the surface roughness and microhardness of nanocomposites. Oper Dent.

[14] Wheeler J, Deb S, Millar BJ (2020). Evaluation of the effects of polishing systems on surface roughness and morphology of dental composite resin. Br Dent J.

[15] Patel B, Chhabra N, Jain D (2016). Effect of different polishing systems on the surface roughness of nano-hybrid composites. J Conserv Dent.

[16] Türkün L (2004). Effect of re-use of a disposable micro-polisher on the surface of a microhybrid resin composite. Am J Dent.

[17] (2017). Bonded abrasives - Determination of grain size distribution. International Organization for Standardization.

[18] Takanashi E, Kishikawa R, Ikeda M, Inai N, Otsuki M, Foxton RM (2008). Influence of abrasive particle size on surface properties of flowable composites. Dent Mater J.

[19] Güntekin N, Tunçdemir AR (2024). Comparison of volumetric loss and surface roughness of composite dental restorations obtained by additive and subtractive manufacturing methods. Heliyon.

[20] Alharbi G, Al Nahedh HN, Al-Saud LM, Shono N, Maawadh A (2024). Effect of different finishing and polishing systems on surface properties of universal single shade resin-based composites. BMC Oral Health.

[21] Marghalani HY (2010). Effect of finishing/polishing systems on the surface roughness of novel posterior composites. J Esthet Restor Dent.

[22] Dennis T, Zoltie T, Wood D, Altaie A (2021). Reduced-step composite polishing systems - a new gold standard?. J Dent.

[23] Jaramillo-Cartagena R, López-Galeano EJ, Latorre-Correa F, Agudelo-Suárez AA (2022). Effect of polishing systems on the surface roughness of nano-hybrid and nano-filling composite resins: A systematic review. Dent J (Basel).

[24] Soliman HAN, Elkholany NR, Hamama HH, El-Sharkawy FM, Mahmoud SH, Comisi JC (2021). Effect of different polishing systems on the surface roughness and gloss of novel nanohybrid resin composites. Eur J Dent.

[25] Altınışık H, Özyurt E (2024). Effect of different polishing systems on surface roughness and gloss values of single-shade resin composites. BMC Oral Health.

[26] Uribe-Hernández L, Latorre-Correa F, Perea-Lowery L, Ardila CM (2024). Effect of preheating and curing lamp distance on the degree of conversion of four nanohybrid resins: An in vitro study. J Clin Exp Dent.

[27] Pineda-Vélez E, Yadalam PK, Ardila CM (2024). Efficacy of the finite element analysis in assessing the effects of light curing on the mechanical properties of direct restorative composites: A systematic review. J Clin Exp Dent.

